# Association between Body Mass Index and Brain Health in Adults: A 16-Year Population-Based Cohort and Mendelian Randomization Study

**DOI:** 10.34133/hds.0087

**Published:** 2024-03-15

**Authors:** Han Lv, Na Zeng, Mengyi Li, Jing Sun, Ning Wu, Mingze Xu, Qian Chen, Xinyu Zhao, Shuohua Chen, Wenjuan Liu, Xiaoshuai Li, Pengfei Zhao, Max Wintermark, Ying Hui, Jing Li, Shouling Wu, Zhenchang Wang

**Affiliations:** ^1^ Department of Radiology, Beijing Friendship Hospital, Capital Medical University, Beijing 100050, China.; ^2^ Peking University School of Public Health, Beijing 100191, China.; ^3^ Department of General Surgery, Beijing Friendship Hospital, Capital Medical University, Beijing 100050, China.; ^4^ Department of Medical Imaging Technology, Capital Medical University Yanjing College, Beijing 101300, China.; ^5^ Center for MRI Research, Peking University Academy for Advanced Interdisciplinary Studies, Beijing 100871, China.; ^6^ Clinical Epidemiology and Evidence-based Medicine Unit, Beijing Friendship Hospital, Capital Medical University, Beijing 100050, China.; ^7^ Department of Cardiology, Kailuan General Hospital, Hebei, Tangshan 063000, China.; ^8^ Department of Neuroradiology, The University of Texas MD Anderson Cancer Center, Houston, TX 78701, USA.; ^9^ Department of Radiology, Kailuan General Hospital, Hebei, Tangshan 063000, China.; ^10^ Department of Radiology, Beijing Tsinghua Changgung Hospital, School of Clinical Medicine, Tsinghua University, Beijing, China.

## Abstract

**Background:** The cumulative effect of body mass index (BMI) on brain health remains ill-defined. The effects of overweight on brain health across different age groups need clarification. We analyzed the effect of cumulative BMI on neuroimaging features of brain health in adults of different ages. **Methods:** This study was based on a multicenter, community-based cohort study. We modeled the trajectories of BMI over 16 years to evaluate cumulative exposure. Multimodality neuroimaging data were collected once for volumetric measurements of the brain macrostructure, white matter hyperintensity (WMH), and brain microstructure. We used a generalized linear model to evaluate the association between cumulative BMI and neuroimaging features. Two-sample Mendelian randomization analysis was performed using summary level of BMI genetic data from 681,275 individuals and neuroimaging genetic data from 33,224 individuals to analyze the causal relationships. **Results:** Clinical and neuroimaging data were obtained from 1,074 adults (25 to 83 years). For adults aged under 45 years, brain volume differences in participants with a cumulative BMI of >26.2 kg/m^2^ corresponded to 12.0 years [95% confidence interval (CI), 3.0 to 20.0] of brain aging. Differences in WMH were statistically substantial for participants aged over 60 years, with a 6.0-ml (95% CI, 1.5 to 10.5) larger volume. Genetic analysis indicated causal relationships between high BMI and smaller gray matter and higher fractional anisotropy in projection fibers. **Conclusion:** High cumulative BMI is associated with smaller brain volume, larger volume of white matter lesions, and abnormal microstructural integrity. Adults younger than 45 years are suggested to maintain their BMI below 26.2 kg/m^2^ for better brain health. **Trial Registration:** This study was registered on clinicaltrials.gov (Clinical Indicators and Brain Image Data: A Cohort Study Based on Kailuan Cohort; No. NCT05453877; https://clinicaltrials.gov/ct2/show/NCT05453877).

## Introduction

Brain health is an evolving concept that is receiving more attention not only from academia but also from wider society. Defined as the preservation of optimal brain integrity, mental function, and cognitive function and the absence of overt neurological disorders [1], brain health may be affected by various conditions.

The health burden of overweight and obesity has substantially increased over the last two decades [2]. Characterized by high body mass index (BMI), overweight and obesity are associated with poorer brain health, resulting in accelerated cognitive decline and dementia [3]. Magnetic resonance imaging (MRI)-based neuroimaging features allow for the objective assessment of brain health. Several cross-sectional studies that acquired BMI and neuroimaging data simultaneously found that individuals with high BMI had smaller brain volumes [4–6] and altered white matter (WM) integrity [7].

Overweight and obesity may require months of energy accumulation. The cumulative effect of BMI, rather than a cross-sectional value of BMI, is more rational to reflect the burden of overweight or obesity. However, the cumulative effect of BMI on brain health has not been adequately studied. Several previous studies analyzed the effect of BMI on features of the brain, but the number of neuroimaging features was limited [4–7]. In addition, knowledge is lacking about the effect of overweight or obesity on brain health in different age groups. A reference value of BMI at different ages would be helpful in promoting brain health. More importantly, BMI and brain health may be associated with various factors in the real world. The associations between BMI and brain health need to be validated when excluding unmeasured confounding factors. Determining a causal relationship is preferred.

In this study, we hypothesized that high cumulative BMI exposure, especially during specific periods in the life course, negatively affects brain health in various ways. The primary objective of this study was to investigate the effect of cumulative BMI on various neuroimaging features of brain health. The secondary objective was to further investigate causal relationships between BMI and neuroimaging features using genetic information [8]. Our study will provide a new strategic direction in brain health protection.

## Methods

### Population-based study

#### Design and participants

This study was conducted as part of the Kailuan Study (KLS), a multicenter, long-term follow-up, community-based cohort consisting of an adult population in Kailuan, Hebei Province, China, since 2006 [9,10]. The included participants were mainly Kailuan (Group) Co. Ltd. employees and regional residents.

Since 2020, participants have been enrolled in a subset of the KLS, called the Multimodality Medical Imaging Study Based on Kailuan Study (META-KLS), which specifically focused on the analysis of neuroimaging. A detailed description of the META-KLS has been published [11]. These participants were recruited using the same method as in the KLS. As of September 2022, a total of 1,195 participants had completed neuroimaging examination once. This research is based on the META-KLS dataset. Participants in the META-KLS are characterized by a long-term follow-up since 2006 and have undergone one neuroimaging examination since 2020.

#### Measurements of clinical features

Demographic questionnaires, clinical information, and laboratory examinations were prospectively collected every 2 years from 2006 to 2018, according to standardized protocols from 11 local hospitals [9,10]. Measurements of clinical features are described in the Supplementary Materials and the previously published protocol [11].

#### Neuroimaging data acquisition

Neuroimaging data were acquired using a 3.0 Tesla MRI scanner (General Electric 750 W, Milwaukee, WI, USA) from 2020 to 2022. The scanning sequences included three-dimensional brain volume (3D-BRAVO) for brain macrostructural volume analysis based on high-resolution T1-weighted imaging (T1WI), diffusion tensor imaging (DTI) for brain microstructural integrity analysis, 3D fluid-attenuated inversion recovery (FLAIR) for white matter hyperintensity (WMH) analysis, and T2-weighted images and diffusion-weighted images for the determination of neuro-dysplasia and stroke. Details of the neuroimaging acquisition parameters are listed in Table S1.

#### Inclusion and exclusion criteria

**Fig. 1.**
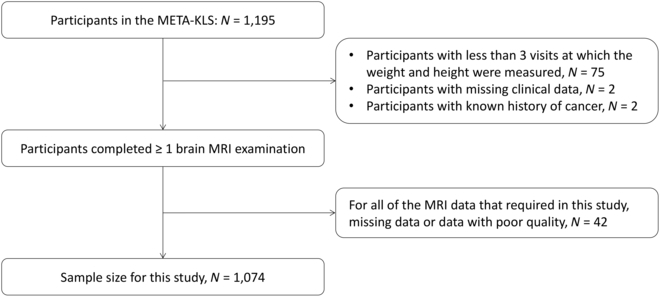
Flowchart of included participants.

All participants in the META-KLS (*N* = 1,195) were initially enrolled in this study. Then, we included participants who met all of the following inclusion criteria: (a) visited the hospital ≥3 times, during which clinical data were collected; (b) completed one brain MRI examination from 2020 to 2022; and (c) without clinically diagnosed or self-reported dementia or mental disorders. The exclusion criteria were as follows: (a) incomplete neuroimaging data; (b) MRI scans with poor quality that were invalid for data analysis, as determined automatically before imaging processing; and (c) a diagnosis of neuro-dysplasia, stroke, or cancer history. The flowchart of the study is shown in Fig. 1.

#### Cumulative BMI exposure based on the longitudinal dataset

The primary exposure of interest was the cumulative BMI over 16 years, which was calculated as the sum of BMI (area under the curve during follow-up, expressed as kg/m^2^ × years) divided by the follow-up duration (years). The cumulative index reflects the cumulative burden of the risk factor, which is more precise than cross-sectional measurements. Meanwhile, the cumulative index also has the same unit (kg/m^2^) and results similar to those of one-time BMI measurements, which are more easily comprehensible [12].

#### Neuroimaging data processing

A standardized and validated neuroimaging data processing workflow was developed to enable an accurate, objective, high-efficiency, and reproducible analysis. Detailed descriptions of the neuroimaging data processing can be found in the published protocol [11]. Neuroimaging features, including relative volume of brain macrostructure, relative volume of WMH, and brain microstructural integrity, were defined as the outcome of this study.

The brain macrostructural volume was calculated based on 3D-BRAVO T1WI. The total intracranial volume (TIV) and volume of supratentorial intracranial structures, including gray matter (GM), WM, and cerebrospinal fluid (CSF), were quantified using an automatic pipeline based on the Statistical Parametric Mapping software (Wellcome Trust Centre for Neuroimaging). The cerebral parenchyma was defined as the sum of GM and WM. We also calculated hippocampal volumes according to the automatic segmentation results of the AAL_90 atlas. The voxel levels of macrostructural volume were analyzed in subregions among groups using the FMRIB Software Library (FSL) v6.0.

We calculated the volume of WMH, periventricular white matter hyperintensity (PWMH), and deep white matter hyperintensity (DWMH) at the voxel level based on 3D FLAIR sequences using the Lesion Prediction Algorithm [13]. The volumetric results were calculated as a percentage of TIV to derive the relative volume of the brain structure.

We measured the markers that reflected brain microstructural integrity using DTI. Specifically, fractional anisotropy (FA) indicates the coherence of the water molecule diffusion direction. Cerebral mean diffusivity (MD) measures the average diffusion unrelated to tissue-based directionality [14,15]. Voxel-wise tract-based spatial statistical analyses were conducted to test between-group differences in the FA and MD maps using FSL v6.0.

#### Statistical analyses

For the population-based study, we used a generalized linear model to evaluate the association between cumulative BMI and neuroimaging features. To analyze the dose-response relationship between BMI and neuroimaging features, we categorized BMI values into tertiles (with cutoff values set at 23.55 and 26.20 kg/m^2^), using the category with the lowest BMI as reference. Model 1 was adjusted for age, sex, and lifestyle factors, including smoking, alcohol consumption, and physical activity. Model 2 added cardiovascular risk factors as additional covariates (full adjustment), including systolic blood pressure, history of diabetes, triglycerides, high-density lipoprotein, and low-density lipoprotein. The average volumes of the brain structures were calculated. Results were further adjusted to exclude the possible impact of cognition, as evaluated by the Montreal Cognitive Assessment. To validate the results, we also tried different methods to classify the groups of BMI: (a) exclude participants with a BMI lower than 18.5 kg/m^2^ and (b) set the cutoff values as 24.0 and 28.0 kg/m^2^ for three groups, according to the recommended criteria for overweight and obesity in Chinese people.

For the macrostructural volume and microstructural integrity of brain subregions at the voxel level, statistical analysis was performed with a general linear model using FSL v6.0. To correct for multiple comparisons, significant clusters were formed by employing threshold-free cluster enhancement (TFCE) via 5,000 permutations. For macrostructural volume, we reported regions with *P*_TFCE_ < 0.01 with >1,000 voxels, with age, sex, and TIV as covariates. For microstructural integrity, clusters with *P*_TFCE_ < 0.05 and those with probabilities of an affected tract threshold >3% were reported, with age and sex as covariates. Probabilities of affected tracts were calculated in different labeled regions within the XTRACT HCP Probabilistic Tract Atlases or Harvard-Oxford Subcortical Structural Atlas.

We calculated the years of brain aging based on the regressing volume of cerebral parenchyma on age [16] to estimate the time necessary for participants with a high BMI to achieve the same cerebral parenchyma volume as those with a low BMI (years of brain aging). For participants in different age groups, the slope (β) was −0.10% for age <45 years, −0.20% for age 45 to 60 years, and −0.40% for age >60 years. The results were further stratified by age and sex to examine the effect of BMI on neuroimaging features in the different groups. The cutoff ages were set at 45 and 60 years. The significance threshold was set at *P* < 0.05, determined by the least significant difference considered statistically significant after correcting for multiple testing.

### Mendelian randomization analysis

Mendelian randomization analysis allows for the unbiased detection of the causal effects of risk factors (higher BMI) on outcomes (volumes of cerebral parenchyma, GM, WM, CSF, bilateral hippocampi, WMH, FA, and MD) because genetic variations are randomly allocated at conception.

The genetic variants used in the study design must meet three assumptions: (a) the genetic variants used as instrumental variables are strongly associated with the risk factor [higher BMI per 1 standard deviation (SD)], (b) instrumental variables should not be associated with other confounders, and (c) instrumental variables are associated with the outcome (neuroimaging features) only through the investigated risk factor [17,18].

We applied two-sample Mendelian randomization analysis to test the causal association between BMI and neuroimaging features. We used genome-wide association study (GWAS) datasets of neuroimaging-derived and BMI-related phenotypes. GWAS summary statistics on BMI were extracted from a meta-analysis study combining the Genetic Investigation of ANthropometric Traits consortium and United Kingdom Biobank, including 681,275 individuals of European ancestry [19]. In this study, 941 near-independent single-nucleotide polymorphisms (SNPs) associated with BMI were identified at a genome-wide significance threshold of *P* < 5 × 10^−8^.

The GWAS dataset of neuroimaging-derived phenotypes was obtained from the Oxford Brain Imaging Genetics web server (https://open.win.ox.ac.uk/ukbiobank/big40/), which included GWAS summary statistics of 33,224 individuals from the United Kingdom Biobank. The discovery sample size was 22,138 with a genome-wide significance set at *P* < 5 × 10^−8^. The replication sample size was 11,086 with *P* < 0.05, without linkage disequilibrium [20]. Linkage disequilibrium is the nonrandom association of alleles at different loci in a given population. Loci are said to be in linkage disequilibrium when the frequency of association of their different alleles is higher or lower than expected if the loci were independent and randomly associated. The analysis of FA and MD focused on skeletonized WM tracts based on the results of the voxel-wise analysis of microstructural integrity in the population-based study.

#### Statistical analysis

The effect estimates of genetically predicted higher BMI on neuroimaging-derived phenotypes are presented as β with their 95% confidence interval (CIs) per 1-SD increment of BMI. Genetic variants served as instrumental variables when reaching the genome-wide significance of *P* < 5 × 10^−8^ and were then clumped to eliminate linkage disequilibrium (*r*^2^ = 0.001) and genomic regions (clump window: 10,000 kilobases). Furthermore, all SNPs in the summary statistics downloads had a minor allele frequency of ≥0.001. As the most widely applied method for Mendelian randomization analysis, the inverse-variance weighted (IVW) method can provide robust causal estimates in the absence of directional pleiotropy [21]. Pleiotropy occurs when one gene influences two or more seemingly unrelated phenotypic traits. In this study, IVW was performed to estimate the causal effect of BMI on neuroimaging features. Statistical significance was set at *P* < 0.05, determined by the least significant difference considered statistically significant after correcting for multiple testing.

We used the Mendelian randomization-Egger (MR-Egger) intercept test and Cochran’s *Q* statistic for pleiotropy and heterogeneity assessment of each instrument in IVW analysis [22,23]. Genetic heterogeneity occurs through the production of single or similar phenotypes through different genetic mechanisms. The Mendelian Randomization Pleiotropy RESidual Sum and Outlier test was performed to detect and rule out horizontal pleiotropic outlier SNPs [24]. We conducted a sensitivity analysis to validate the robustness of the results using five different Mendelian randomization methods: MR-Egger regression, weighted median, simple mode, weighted mode, and leave-one-out.

This study followed the Strengthening the Reporting of Observational Studies in Epidemiology (STROBE) [25] and STROBE Mendelian randomization reporting guidelines [26]. The IBM SPSS Statistics for Windows version 26.0 (IBM Corp., Armonk, NY, USA) and R 4.2.1 (R Development Core Team) were used for statistical analyses.

## Results

### Population-based study

A total of 1,074 participants, aged 25 to 83 years, were eligible for this study; 761 (70.9%) were followed up for 16 years since 2006. Each participant had a median of five visits with clinical information and laboratory examinations. The mean (SD) age was 55.2 (11.6) years, and 473 (44.0%) participants were female. Table 1 lists the participants’ clinical characteristics and neuroimaging features according to age. Compared with young adults, middle-aged participants were more likely to have smoking and drinking habits, an adverse cardiovascular risk profile, a smaller brain volume, abnormal microstructural integrity, and a larger volume of WMH.

**Table 1.** Demographic and clinical characteristics of participants

	All	Age < 45 years	45 years ≤ age < 60 years	Age ≥ 60 years	*P* ^a^
Demographics	*N* = 1,074	*N* = 227	*N* = 459	*N* = 388	
Age, mean (SD), years^b^	55.2 (11.6)	38.5 (4.4)	53.1 (4.2)	67.4 (4.7)	<0.001
Female, no. (%)	473 (44.0)	132 (58.1)	206 (44.9)	135 (34.8)	<0.001
Cumulative BMI, mean (SD), kg/m^2^	25.0 (3.0)	24.8 (3.5)	25.0 (2.8)	25.2 (2.8)	0.343
Cumulative BMI, no. (%), kg/m^2^					
<18.5	5 (0.5)	1 (0.4)	1 (0.2)	3 (0.8)	0.265
18.5–22.9	276 (25.7)	76 (33.5)	118 (25.7)	85 (21.9)	0.468
23.0–24.9	270 (25.1)	48 (21.1)	110 (24.0)	110 (28.4)	0.468
25.0–29.9	460 (42.8)	84 (37.0)	207 (45.1)	168 (43.3)	0.457
30.0–34.9	63 (5.9)	18 (7.9)	23 (5.0)	22 (5.7)	0.433
≥35.0	0	0	0	0	N/A
Lifestyle factors, no. (%)					
Smoking habits					
Never	647 (60.2)	172 (75.8)	279 (60.8)	196 (50.5)	<0.001
Past	205 (19.1)	22 (9.7)	77 (16.8)	106 (27.3)	
Current	222 (20.7)	33 (14.5)	103 (22.4)	86 (22.2)	
Alcohol consumption					
Never	637 (59.3)	170 (74.9)	237 (51.6)	230 (59.3)	<0.001
Past	144 (13.4)	19 (8.4)	67 (14.6)	58 (14.9)	
Current	293 (27.3)	38 (16.7)	155 (33.8)	100 (25.8)	
Physical activity					
Sometimes or seldom	899 (83.7)	198 (83.3)	393 (85.6)	317 (81.7)	0.300
Usually	175 (16.3)	38 (16.7)	66 (14.4)	71 (18.3)	
Cardiovascular risk factors					
History of hypertension, no. (%)	723 (67.3)	124 (54.6)	307 (66.9)	292 (75.3)	<0.001
Blood pressure, mean (SD), mm Hg					
Systolic	126.3 (13.2)	120.6 (10.7)	124.9 (12.1)	131.3 (13.9)	<0.001
Diastolic	81.1 (8.0)	78.8 (8.0)	81.3 (8.3)	82.1 (7.5)	<0.001
History of diabetes, no. (%)	208 (19.4)	20 (8.8)	75 (16.3)	113 (29.1)	<0.001
Cholesterol, mM					
Triglyceride, median (IQR)	1.5 (1.1, 2.1)	1.3 (1.0, 1.9)	1.5 (1.0, 2.2)	1.5 (1.2, 2.0)	0.028
High-density lipoprotein, mean (SD)	1.5 (0.3)	1.5 (0.3)	1.5 (0.3)	1.5 (0.3)	0.243
Low-density lipoprotein, mean (SD)	2.7 (0.6)	2.5 (0.5)	2.6 (0.6)	2.8 (0.7)	<0.001
Neuroimaging features					
TIV, mean (SD), ml	1,498.9 (139.4)	1,490.5 (158.8)	1,500.8 (137.5)	1,501.7 (128.6)	0.601
Relative brain macrostructural volume, mean (SD), % of TIV	*N* = 1,011	*N* = 221	*N* = 430	*N* = 360	
Cerebral parenchyma	73.3 (4.1)	76.9 (2.9)	74.3 (3.0)	69.9 (3.5)	<0.001
Gray matter	39.9 (2.7)	42.4 (2.3)	40.3 (2.1)	37.9 (2.2)	<0.001
White matter	33.4 (2.1)	34.5 (1.6)	33.9 (1.7)	32.0 (2.1)	<0.001
Cerebrospinal fluid	26.5 (4.1)	23.0 (2.8)	25.6 (2.9)	29.8 (3.4)	<0.001
Hippocampus	0.24 (0.02)	0.26 (0.02)	0.25 (0.02)	0.23 (0.03)	<0.001
Brain microstructural integrity, mean (SD)	*N* = 1,042	*N* = 222	*N* = 447	*N* = 373	
Fractional anisotropy	0.46 (0.03)	0.47 (0.01)	0.46 (0.02)	0.44 (0.03)	<0.001
Mean diffusivity, 10^−3^ mm^2^/s	0.82 (0.03)	0.80 (0.02)	0.81 (0.03)	0.85 (0.04)	<0.001
Relative white matter hyperintensity volume, median (IQR), % of TIV	*N* = 986	*N* = 216	*N* = 422	*N* = 348	
White matter hyperintensity	0.23 (0.11–0.55)	0.10 (0.04, 0.18)	0.20 (0.11, 0.36)	0.60 (0.30, 1.12)	<0.001
Periventricular white matter hyperintensity	0.14 (0.06–0.33)	0.07 (0.03, 0.11)	0.12 (0.06, 0.21)	0.38 (0.17, 0.67)	<0.001
Deep white matter hyperintensity volume	0.09 (0.03–0.22)	0.03 (0, 0.07)	0.07 (0.04, 0.14)	0.22 (0.10, 0.47)	<0.001
Cognitive assessment					
MoCA (Montreal Cognitive Assessment)	24.55 (3.81)	27.0 (2.3)	25.3 (3.1)	22.3 (4.1)	<0.001

BMI, body mass index; TIV, total intracranial volume; IQR, interquartile range; N, number

Values are presented as mean (SD), median (IQR), or no. (%).

^a^ Differences among age groups. Comparisons were based on analysis of variance, Kruskal–Wallis test, or chi-square test.

^b^ Age (in years) was calculated at the time of MRI acquisition.

**Fig. 2.**
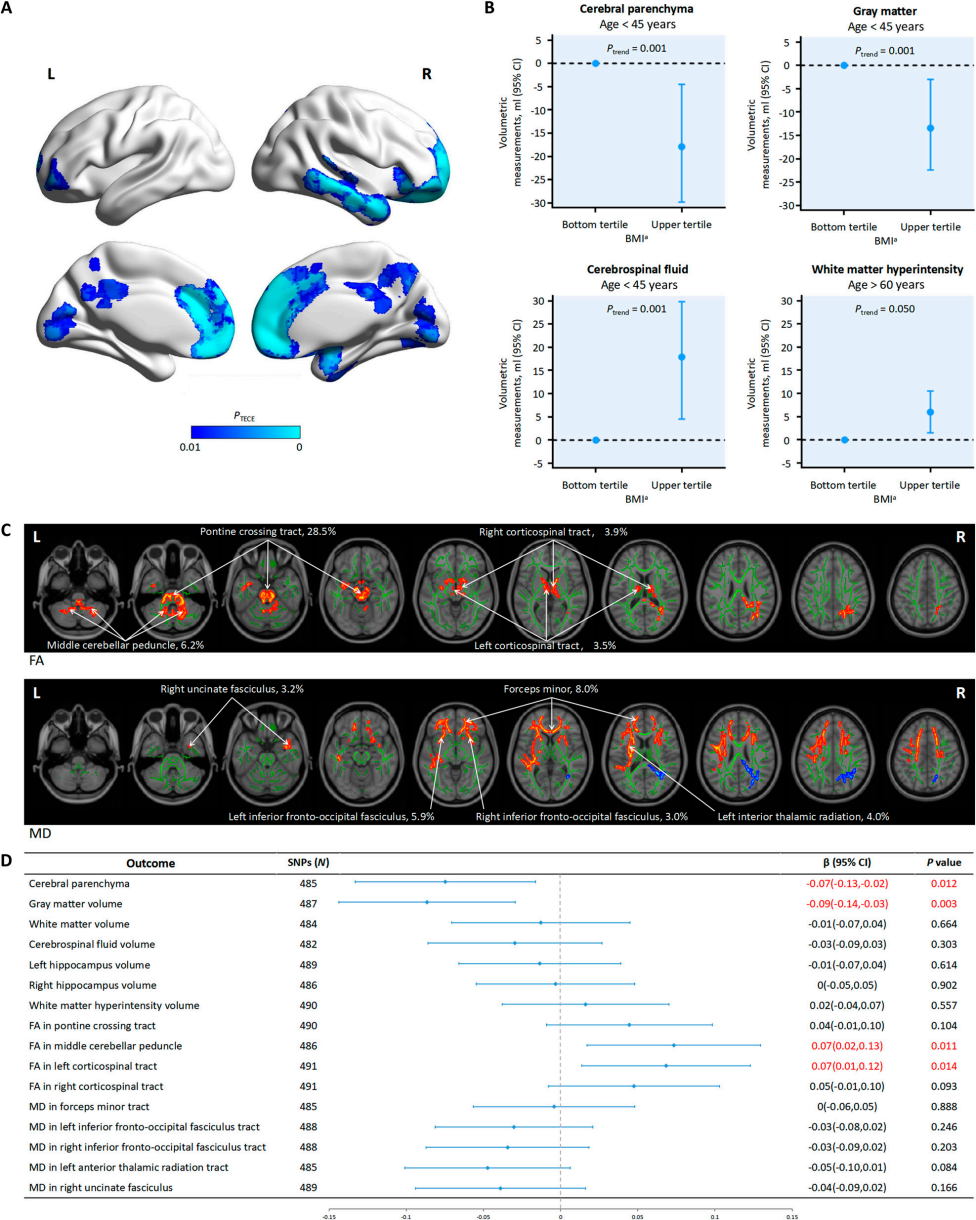
Association of high BMI with neuroimaging features. (A) High BMI is associated with a smaller GM volume. Results are based on a comparison of upper versus bottom tertiles at the voxel level. Regions with *P*_TFCE_ < 0.01 are reported, with age, sex, and TIV as covariates. Brain atrophy is predominantly in the frontal lobe, temporal lobe, and anterior cingulate gyrus. Cold color represents a smaller value. (B) Association of BMI with volume of the cerebral parenchyma, GM, and white matter hyperintensity in different age groups. The associations of high BMI and a relative volume of cerebral parenchyma, GM, and CSF are significant in young adults. High BMI was associated with larger relative volumes of white matter hyperintensity in participants over 60 years of age. ^a^Cumulative BMI <23.55 kg/m^2^ (bottom tertile) and >26.20 kg/m^2^ (upper tertile). (C) Association of BMI with microstructural integrity. Results are based on a comparison of upper versus bottom tertiles at the voxel level. Clusters with *P*_TFCE_ < 0.05 and those with probabilities of affected tracts threshold >3% are reported, with age and sex as covariates. Results are labeled as tract, probabilities (%). Hot and cold colors represent higher and lower values, respectively. (D) Mendelian randomization association of genetic determinants of high BMI with neuroimaging features. Forest plot presenting causal effect estimates for genetically predicted BMI on neuroimaging features. The results were derived from the IVW method and presented as β (95% confidence intervals). Red rows represent statistically significant associations at *P* < 0.05. L, left; R, right.

After full adjustment, high cumulative BMI (top versus bottom tertiles) was associated with a smaller relative volume of the cerebral parenchyma and GM in the general population [β = −0.60; 95% CI, −1.10 to −0.20; *P* for trend (*P*_trend_) = 0.026 and β = −0.50; 95% CI, −0.80 to −0.20; *P*_trend_ = 0.003, respectively] (Table 2). High cumulative BMI was also associated with a larger relative volume of CSF (β = 0.60; 95% CI, 0.20 to 1.10; *P*_trend_ = 0.024). Volumetric results corresponded to −8.99 ml (95% CI, −16.49 to −3.00) in the cerebral parenchyma, −7.49 ml (95% CI, −11.99 to −3.00) in the GM, and 8.99 ml (95% CI, 3.00 to 16.49) in CSF. Voxel-wise analysis demonstrated significant GM atrophy, predominantly in the frontal lobe, temporal lobe, and anterior cingulate gyrus (Fig. 2A). No significant associations were observed between BMI and the relative volume of WM or the hippocampus.

**Table 2.** Association of cumulative BMI with brain macrostructural volume, brain microstructural integrity, and white matter hyperintensity

Neuroimaging features	Cumulative BMI, kg/m^2^			*P* valuefor trend
	Low	Medium	High	
	(<23.56)	(23.56–26.20)	(>26.20)	
Cumulative BMI, mean (SD), kg/m^2^	21.8 (1.3)*N* = 357	24.9 (0.8)*N* = 353	28.3 (1.7)*N* = 364	
Relative brain macrostructural volume, % of TIV				
Cerebral parenchyma				
Model 1	0 (ref)	−0.30 (−0.70 to 0.10)	−0.90 (−1.30 to −0.50)	<0.001
Model 2	0 (ref)	−0.20 (−0.60 to 0.20)	−0.60 (−1.10 to −0.20)	0.026
Gray matter				
Model 1	0 (ref)	−0.40 (−0.60 to −0.10)	−0.70 (−1.00 to −0.40)	<0.001
Model 2	0 (ref)	−0.30 (−0.60 to 0.00)	−0.50 (−0.80 to −0.20)	0.003
White matter				
Model 1	0 (ref)	0 (−0.20 to 0.30)	−0.20 (−0.50 to 0.00)	0.089
Model 2	0 (ref)	0.10 (−0.20 to 0.40)	−0.10 (−0.40 to 0.20)	0.434
Cerebrospinal fluid				
Model 1	0 (ref)	0.30 (−0.10 to 0.70)	0.90 (0.50 to 1.30)	<0.001
Model 2	0 (ref)	0.20 (−0.20 to 0.70)	0.60 (0.20 to 1.10)	0.024
Hippocampus				
Model 1	0 (ref)	−2.4E−3 (−3.3E−3 to 2.8E−3)	−2.1E−03 (−5.2E−3 to 9.7E−4)	0.326
Model 2	0 (ref)	−1.8E−4 (−3.3E−3 to 2.9E−3)	−1.7E−3 (−5.1E−3 to 1.7E−3)	0.546
Brain microstructural integrity				
Fractional anisotropy				
Model 1	0 (ref)	0.002 (−0.001 to 0.005)	0 (−0.003 to 0.004)	0.445
Model 2	0 (ref)	0.003 (−0.001 to 0.006)	0.002 (−0.002 to 0.006)	0.273
Mean diffusivity, 10^−3^ mm^2^/s				
Model 1	0 (ref)	−0.003 (−0.007 to 0.002)	5.0E−4 (−4.3E−3 to 5.4E−3)	0.367
Model 2	0 (ref)	−0.004 (−0.009 to 0.001)	−0.003 (−0.009 to 0.002)	0.233
Relative white matter hyperintensity volume, % of TIV				
White matter hyperintensity				
Model 1	0 (ref)	0 (−0.10 to 0.20)	0.30 (0.20 to 0.40)	<0.001
Model 2	0 (ref)	0 (−0.10 to 0.10)	0.20 (0.00 to 0.30)	0.007
Periventricular white matter hyperintensity				
Model 1	0 (ref)	0 (0.00 to 0.10)	0.10 (0.10 to 0.20)	<0.001
Model 2	0 (ref)	0.01 (0.00 to 0.10)	0.10 (0.00 to 0.10)	0.011
Deep white matter hyperintensity				
Model 1	0 (ref)	0 (−0.10 to 0.10)	0.20 (0.10 to 0.20)	<0.001
Model 2	0 (ref)	0 (−0.10 to 0.10)	0.10 (0.01 to 0.20)	0.013

BMI, body mass index; TIV, total intracranial volume; IQR, interquartile range; CI, confidence interval

Model 1: Adjusted for age, sex, and lifestyle factors including smoking, alcohol consumption, and physical activity. Model 2: Model 1 additionally adjusted for cardiovascular risk factors, including systolic blood pressure, history of diabetes, triglycerides, high-density lipoprotein, and low-density lipoprotein. Results are presented as β (95% CI). Values are presented as mean (SD), median (IQR), or no. (%).

Regarding the results in different age groups, the associations of high cumulative BMI and relative volumes of the cerebral parenchyma, GM, and CSF were only significant in young adults after full adjustment (β = −1.20; 95% CI, −2.00 to −0.30; β = −0.90; 95% CI, −1.50 to −0.20; and β = 1.20; 95% CI, 0.30 to 2.00, respectively) (Table 3). Volumetric results corresponded to −17.9 ml (95% CI, −29.8 to −4.5) in the cerebral parenchyma, −13.4 ml (95% CI, −22.4 to −3.0) in the GM, and 17.9 ml (95% CI, 4.5 to 29.8) in CSF (Fig. 2B). The results corresponded to 12.0 years (95% CI, 3.0 to 20.0) of brain aging in young adults with a cumulative BMI over 26.2 kg/m^2^.

**Table 3.** Association of cumulative BMI with brain macrostructural volume, brain microstructural integrity, and white matter hyperintensity stratified by age

Neuroimaging features	Age < 45 years				45 years ≤ age < 60 years				Age ≥ 60 years			
	Low(<23.56)	Medium(23.56–26.20)	High(>26.20)	*P* valuefor trend	Low(<23.56)	Medium(23.56–26.20)	High(>26.20)	*P* valuefor trend	Low(<23.56)	Medium(23.56–26.20)	High(>26.20)	*P* valuefor trend
Cumulative BMI, mean (SD), kg/m^2^	21.5 (1.3)*N* = 97	24.8 (0.8)*N* = 51	28.8 (1.9)*N* = 79		21.8 (1.2)*N* = 146	24.9 (0.8)*N* = 160	28.1 (1.7)*N* = 153		22.1 (1.3)*N* = 114	24.8 (0.8)*N* = 142	28.2 (1.6)*N* = 132	
Relative brain macrostructural volume, % of TIV												
Cerebral parenchyma												
Model 1	0 (ref)	0.60 (−0.30 to 1.40)	−1.10 (−1.90 to −0.30)	<0.001	0 (ref)	−0.70 (−1.30 to −0.10)	−0.80 (−1.40 to −0.20)	0.022	0 (ref)	−0.60 (−1.30 to 0.10)	−1.00 (−1.70 to −0.30)	0.024
Model 2	0 (ref)	0.50 (−0.40 to 1.30)	−1.20 (−2.00 to −0.30)	0.001	0 (ref)	−0.60 (−1.20 to 0.00)	−0.50 (−1.20 to 0.20)	0.140	0 (ref)	−0.50 (−1.20 to 0.20)	−0.60 (−1.40 to 0.20)	0.268
Gray matter												
Model 1	0 (ref)	0.40 (−0.20 to 1.00)	−0.80 (−1.40 to −0.20)	<0.001	0 (ref)	−0.50 (−0.90 to −0.10)	−0.70 (−1.10 to −0.30)	0.003	0 (ref)	−0.60 (−1.00 to −0.10)	−0.50 (−1.00 to −0.10)	0.028
Model 2	0 (ref)	0.40 (−0.20 to 1.00)	−0.90 (−1.50 to −0.20)	0.001	0 (ref)	−0.50 (−0.90 to −0.10)	−0.50 (−1.00 to 0.00)	0.055	0 (ref)	−0.50 (−1.00 to −0.10)	−0.30 (−0.80 to 0.20)	0.087
White matter												
Model 1	0 (ref)	0.10 (−0.40 to 0.70)	−0.30 (−0.80 to 0.20)	0.343	0 (ref)	−0.20 (−0.60 to 0.20)	−0.10 (−0.50 to 0.30)	0.747	0 (ref)	0 (−0.50 to 0.40)	−0.50 (−1.00 to 0.00)	0.070
Model 2	0 (ref)	0.10 (−0.50 to 0.70)	−0.30 (−0.90 to 0.30)	0.370	0 (ref)	−0.10 (−0.50 to 0.30)	0.01 (−0.50 to 0.50)	0.701	0 (ref)	0 (−0.40 to 0.50)	−0.30 (−0.80 to 0.20)	0.375
Cerebrospinal fluid												
Model 1	0 (ref)	−0.60 (−1.40 to 0.30)	1.10 (0.30 to 1.80)	0.001	0 (ref)	0.70 (0.10 to 1.30)	0.80 (0.20 to 1.40)	0.022	0 (ref)	0.60 (−0.10 to 1.30)	1.00 (0.30 to 1.70)	0.022
Model 2	0 (ref)	−0.50 (−1.30 to 0.40)	1.20 (0.30 to 2.00)	0.001	0 (ref)	0.60 (0.00 to 1.20)	0.50 (−0.20 to 1.20)	0.122	0 (ref)	0.50 (−0.20 to 1.20)	0.60 (−0.10 to 1.40)	0.240
Hippocampus												
Model 1	0 (ref)	3.2E−3 (−2.7E−3 to 9.0E−3)	−3.0E−3 (−8.5E−3 to 2.6E−3)	0.140	0 (ref)	−1.9E−3 (−6.1E−3 to 2.3E−3)	−8.5E−4 (−5.2E−3 to 3.6E−3)	0.675	0 (ref)	−2.8E−3 (−8.1E−3 to 2.4E−3)	−3.8E−3 (−9.1E−3 to 1.6E−3)	0.367
Model 2	0 (ref)	2.6E−3 (−3.4E−3 to 8.5E−3)	−3.5E−3 (−9.5E−3 to 2.6E−3)	0.164	0 (ref)	−2.2E−3 (−6.6E−3 to 2.2E−3)	−5.9E−4 (−5.5E−3 to 4.3E−3)	0.548	0 (ref)	−2.1E−3 (−7.4E−3 to 3.2E−3)	−2.3E−3 (−8.1E−3 to 3.6E−3)	0.687
Brain microstructural integrity												
Fractional anisotropy												
Model 1	0 (ref)	0.003 (−0.002 to 0.008)	0.001 (−0.004 to 0.005)	0.376	0 (ref)	−0.001 (−0.005 to 0.003)	0.001 (−0.003 to 0.005)	0.526	0 (ref)	0.002 (−0.006 to 0.01)	−0.003 (−0.011 to 0.005)	0.403
Model 2	0 (ref)	0.003 (−0.002 to 0.008)	−0.001 (−0.006 to 0.004)	0.405	0 (ref)	−0.001 (−0.004 to 0.003)	0.003 (−0.001 to 0.008)	0.122	0 (ref)	0.004 (−0.004 to 0.011)	0 (−0.008 to 0.009)	0.585
Mean diffusivity, 10^−3^ mm^2^/s												
Model 1	0 (ref)	−0.008 (−0.015 to −0.001)	−0.002 (−0.008 to 0.004)	0.076	0 (ref)	0.004 (−0.002 to 0.009)	0.001 (−0.005 to 0.007)	0.430	0 (ref)	−0.001 (−0.011 to 0.009)	0.006 (−0.004 to 0.016)	0.345
Model 2	0 (ref)	−0.007 (−0.014 to 0)	0 (−0.007 to 0.007)	0.108	0 (ref)	0.003 (−0.003 to 0.009)	−0.001 (−0.008 to 0.005)	0.208	0 (ref)	−0.004 (−0.015 to 0.006)	−0.002 (−0.013 to 0.010)	0.678
Relative white matter hyperintensity volume, % of TIV												
White matter hyperintensity												
Model 1	0 (ref)	0.01 (−0.10 to 0.10)	0.10 (0.00 to 0.20)	0.127	0 (ref)	0 (−0.10 to 0.10)	0.20 (0.00 to 0.30)	<0.001	0 (ref)	0.30 (0.00 to 0.50)	0.60 (0.30 to 0.90)	<0.001
Model 2	0 (ref)	0 (−0.10 to 0.10)	0.10 (0.01 to 0.20)	0.085	0 (ref)	−0.10 (−0.20 to 0.00)	0.10 (−0.10 to 0.20)	0.020	0 (ref)	0.20 (−0.10 to 0.50)	0.40 (0.10 to 0.70)	0.050
Periventricular white matter hyperintensity												
Model 1	0 (ref)	0 (0.00 to 0.00)	0 (0.00 to 0.10)	0.002	0 (ref)	0 (0.00 to 0.00)	0.10 (0.00 to 0.10)	0.004	0 (ref)	0.10 (0.00 to 0.20)	0.20 (0.10 to 0.40)	<0.001
Model 2	0 (ref)	0 (0.00 to 0.00)	0.10 (0.00 to 0.10)	0.003	0 (ref)	0 (−0.10 to 0.00)	0 (0.00 to 0.10)	0.135	0 (ref)	0.10 (0.00 to 0.20)	0.20 (0.00 to 0.30)	0.042
Deep white matter hyperintensity												
Model 1	0 (ref)	0 (−0.10 to 0.10)	0 (0.00 to 0.10)	0.493	0 (ref)	0 (−0.10 to 0.00)	0.10 (0.00 to 0.20)	0.001	0 (ref)	0.10 (0.00 to 0.30)	0.30 (0.20 to 0.50)	0.001
Model 2	0 (ref)	0 (−0.10 to 0.10)	0.10 (0.00 to 0.10)	0.339	0 (ref)	−0.10 (−0.10 to 0.01)	0 (−0.10 to 0.10)	0.015	0 (ref)	0.10 (−0.10 to 0.30)	0.20 (0.00 to 0.40)	0.086

BMI, body mass index; TIV, total intracranial volume; CI, confidence interval

Model 1: Adjusted for age, sex, and lifestyle factors including smoking and alcohol consumption and physical activity. Model 2: Model 1 additionally adjusted for cardiovascular risk factors, including systolic blood pressure, history of diabetes, triglycerides, high-density lipoprotein, and low-density lipoprotein. Results are presented as β (95% CI).

High cumulative BMI was associated with a larger relative volume of WMH, PWMH, and DWMH in the general population (β = 0.20; 95% CI, 0.00 to 0.30; *P*_trend_ = 0.007; β = 0.10; 95% CI, 0.00 to 0.10; *P*_trend_ = 0.011; and β = 0.10; 95% CI, 0.01 to 0.20; *P*_trend_ = 0.013, respectively) (Table 2). Volumetric results corresponded to 3.00 ml (95% CI, 0 to 4.47) in WMH, 1.49 ml (95% CI, 0 to 1.49) in PWMH, and 1.49 ml (95% CI, 0.15 to 3.00) in DWMH.

After full adjustment for cardiovascular risk factors, high cumulative BMI was associated with larger relative volumes of WMH and PWMH in participants over 60 years of age (β = 0.40; 95% CI, 0.10 to 0.70; *P*_trend_ = 0.050 and β = 0.20; 95% CI, 0 to 0.30; *P*_trend_ = 0.042, respectively) (Table 3). Volumetric results corresponded to 6.0 ml (95% CI, 1.5 to 10.5) in WMH and 3.0 ml (95% CI, 0 to 4.5) in PWMH. There were no sex differences in the effects of high BMI on the neuroimaging features analyzed above.

After additional adjustment for the results of cognitive evaluation, the main results remained significant (Tables S4 and S5). The results were also stable when we (a) excluded five participants with a cumulative BMI lower than 18.5 kg/m^2^ (Table S6) and (b) set the cutoff values as 24.0 and 28.0 kg/m^2^ for three groups (Table S7).

The brain microstructural integrity did not differ at the whole-brain level. Additional analysis at the voxel level (top versus bottom tertiles) demonstrated a positive association between cumulative BMI and FA in the pontine crossing tract, middle cerebellar peduncle, and projection fibers (bilateral corticospinal tract). Cumulative BMI was also positively associated with MD in callosal fibers (forceps minor), association fibers (bilateral inferior fronto-occipital fasciculus and right uncinate fasciculus), and left anterior thalamic radiation (Fig. 2C). The results were not significant among the age or sex groups.

### Mendelian randomization analysis

In total, 507 genetic variants without linkage disequilibrium were associated with BMI, of which 13 were palindromic SNPs and were excluded from further Mendelian randomization analysis. Cochran’s *Q* statistic indicated heterogeneity of included SNPs in the IVW method, excluding BMI on MD in the left inferior fronto-occipital fasciculus tract. The MR-Egger intercept revealed no significant pleiotropy for these instrumental SNPs (Table S2), thus indicating compliance with the second and third assumptions of the Mendelian randomization analysis.

In the two-sample Mendelian randomization analysis, the IVW method revealed that high BMI was inversely associated with cerebral parenchyma (β = −0.07; 95% CI, −0.13 to −0.02; *P* = 0.012) and GM volumes (β = −0.09; 95% CI, −0.14 to −0.03; *P* = 0.003). Genetically predicted higher BMI was associated with higher FA in the left corticospinal tract (β = 0.07; 95% CI, 0.01 to 0.12; *P* = 0.014) and middle cerebellar peduncle (β = 0.07; 95% CI, 0.02 to 0.13; *P* = 0.011). The association of FA in the right corticospinal tract reached only marginal significance (β = 0.05; 95% CI, −0.01 to 0.10; *P* = 0.09). However, there was no significant difference in the volumes of the WM, WMH, CSF, bilateral hippocampi, FA in the pontine crossing tract, or MD skeletonized tracts (Fig. 2D and Table S3).

For the sensitivity analysis, the weighted median and weighted mode methods presented statistical significance for the causal effect estimation of high BMI on GM volume. The weighted median model also indicated that higher BMI was associated with a higher value in FA in the left corticospinal tract. For other neuroimaging features, the causal relationship with genetically predicted BMI level was not statistically significant in the additional sensitivity analysis (Table S3).

## Discussion

Our study used evidence from a population-based study and genetic analysis to analyze the effect of long-term exposure to high BMI on brain health. The primary findings indicated that high cumulative BMI is detrimental to brain health, as characterized by a decreased volume of the brain macrostructure, altered brain microstructural integrity, and more WM lesions. The secondary results further supported a causal relationship between high BMI and poorer brain health without potential reverse causality bias. An interesting finding was that differences among groups were only significant in young adults. According to the results of the regressing volume of cerebral parenchyma on age, differences corresponded to 12.0 years of brain aging. Thus, for adults younger than 45 years, BMI should be maintained below 26.2 kg/m^2^ for better brain health.

Metrics of subclinical alterations detected on neuroimaging, such as the volume of MRI-defined WMH and microstructural integrity as assessed by DTI, are important features to consider in the evolving definition of optimal brain health [27]. Although the available evidence supporting brain health promotion is incomplete [4,6,28], observational epidemiological evidence for modifiable factors strongly favors addressing brain health through cardiovascular risk modification. Among these, high BMI is a key risk factor that affects brain health and is commonly associated with poor lifestyle, age, hypertension, dyslipidemia, and diabetes mellitus [29]. Hence, the main purpose of our observational study was to provide and clarify an independent causal association between cumulative BMI and neuroimaging features.

Previous cross-sectional studies investigating the effects of high BMI as a risk factor on FA values have yielded inconsistent results [7,28]. Individuals with a high BMI have been shown to have lower FA in widespread WM tracts [7], but such associations with adverse results are due to the nonlongitudinal design. One key innovation of this study is the application of the cumulative BMI index. Cumulative burden measurements, based on models of trajectories using long-term follow-up data, can reduce random errors and provide more precise indices than cross-sectional studies based on data with only one time point [12]. Thus, data from the META-KLS cohort with a long-term follow-up provided results of greater validity compared to those of cross-sectional studies. We suggest that high BMI may be associated with adaptive neuroplasticity or selective neurodegeneration [30], resulting in higher FA values of projection fibers. Other results of this study are in line with those of previous reports, including smaller GM volume in the frontal and temporal regions [31], abnormal microstructural integrity [28], larger WMH volume [32], and nonsignificant associations between BMI and WM or hippocampus volume [33]. These results support the belief that maintaining a favorable body weight leads to the maintenance of brain vitality.

The negative results of brain volume in relatively younger adults are similar to the results of a previous study [16]. Limited high-level evidence could well explain this finding. Further studies are needed to clarify the risk relationships before and after midlife, which may be explained by unmeasured confounding factors other than cardiovascular risk factors associated with BMI.

Several studies have analyzed brain age [34–37]. Generally, these studies focused on developing a model to predict brain age using neuroimaging data, either in healthy adults or in patients. However, our study is different as we did not intend to develop a better method to predict brain age. In light of a previously published article [16,38], we calculated the years of brain aging based on the regressing volume of cerebral parenchyma on age. In this way, the results specifically reflect the changes in this cohort.

The differences in findings between the observational study and Mendelian randomization analysis can be explained as follows. Despite using five Mendelian randomization methods to ensure the reliability of the results, causal relationships were only found between higher BMI and a limited number of neuroimaging features. There may be several reasons. First, previous studies have shown that neuroimaging alterations may be influenced by sleep, education, and other unknown factors [27]. These factors may influence the results of observational studies. Second, we set strict statistical levels in Mendelian randomization analysis for SNP selection to ensure compliance with the first assumption. This may have limited the results.

There were limited number of studies that analyzed the causal association between body weight and neuroimaging metrics. There were several inconsistent findings among the results. As reported by Chen et al. [39], obesity was causally associated with either increased or decreased cortical thickness and cortical surface area. The main reason for the inconsistency finding lies in the diverse neuroimaging metrics that applied. Changes of cortical thickness or cortical surface area do not indicate GM volume alteration. Specifically, our results indicated smaller volume of the GM, but no significant associations were observed in the cortical thickness or cortical surface area [39]. In addition, consistent with previous findings reported by Debette et al. [40], larger waist-to-hip ratio was causally associated with lower GM volume in women. In general, results of the causal conclusion are reliable.

This study has several strengths. First, the enrolled participants were representative. We enrolled a large number of participants with a wide range of ages from the META-KLS, a cohort of enrolled participants from multiple hospitals in Tangshan in the center of the Bohai Sea Gulf region. Therefore, the results are generalizable to northern Chinese people. Second, we highlighted the value of cumulative BMI. Cumulative BMI, rather than one-time measured BMI, reflects the cumulative burden of a risk factor over a long period. Third, neuroimaging features analyzed at the voxel level provided objective and quantitative biomarkers to reflect the state of brain health. Various features reflect brain health from distinct aspects, providing a more comprehensive framework for understanding the state of brain health. We also validated the causal relationship between high BMI and poorer brain health from a genetic perspective. Sensitivity analysis further supported our findings.

Several limitations should be addressed. First, we acquired the neuroimaging data only once. Longitudinally acquired neuroimaging data are needed to analyze changes in brain health over time. Second, there were inherent limitations to the available data, such as those based on body fat measurements. Quantification of adipose tissue may provide a better indication of risk than BMI. Third, evidence is lacking to prove the similarity of genes between people of European and Chinese ancestries. The results of Mendelian randomization analysis should be carefully interpreted since it may not be generalizable to Chinese people. We could not analyze the causality in different age groups, since the raw data of the GWAS were not divided into groups based on different ages with regard to BMI and neuroimaging features.

## Conclusion

High BMI is associated with a smaller brain volume, larger volume of WM lesions, and abnormal microstructural integrity in projection fibers. For adults younger than 45 years, BMI should be maintained below 26.2 kg/m^2^ for better brain health. Our study provides the foundation for a new strategic direction in brain health protection and promotion based on BMI control.

## Data Availability

Clinical data will be available for other research groups whose proposed use of the data has been approved by an independent review committee identified for this purpose. Requests for data should be directed to the principal investigator, Z.W. (cjr.wzhch@vip.163.com).

## References

[1] Wang Y, Pan Y, Li H. What is brain health and why is it important? BMJ. 2020;371: Article m3683. 33037002 10.1136/bmj.m3683 PMC7555053

[2] GBD 2015 Obesity Collaborators, Afshin A, Forouzanfar MH, Reitsma MB, Sur P, Estep K, Lee A, Marczak A, Mokdad AH, Moradi-Lakeh M, et al. Health effects of overweight and obesity in 195 countries over 25 years. N. Engl. J. Med. 2017;377(1):13–27. 28604169 10.1056/NEJMoa1614362 PMC5477817

[3] Deng YT, Li YZ, Huang SY, Ou YN, Zhang W, Chen SD, Zhang YR, Yang L, Dong Q, Feng JF, et al. Association of life course adiposity with risk of incident dementia: A prospective cohort study of 322,336 participants. Mol. Psychiatry. 2022;27(8):3385–3395. 35538193 10.1038/s41380-022-01604-9

[4] Taki Y, Kinomura S, Sato K, Inoue K, Goto R, Okada K, Uchida S, Kawashima R, Fukuda H. Relationship between body mass index and gray matter volume in 1,428 healthy individuals. Obesity (Silver Spring). 2008;16(1):119–124. 18223623 10.1038/oby.2007.4

[5] Raji CA, Ho AJ, Parikshak NN, Becker JT, Lopez OL, Kuller LH, Hua X, Leow AD, Toga AW, Thompson PM. Brain structure and obesity. Hum. Brain Mapp. 2010;31(3):353–364. 19662657 10.1002/hbm.20870 PMC2826530

[6] Hamer M, Batty GD. Association of body mass index and waist-to-hip ratio with brain structure: UK Biobank study. Neurology. 2019;92(6):e594–e600. 30626649 10.1212/WNL.0000000000006879 PMC8093082

[7] Repple J, Opel N, Meinert S, Redlich R, Hahn T, Winter NR, Kaehler C, Emden D, Leenings R, Grotegerd D, et al. Elevated body-mass index is associated with reduced white matter integrity in two large independent cohorts. Psychoneuroendocrinology. 2018;91:179–185. 29571075 10.1016/j.psyneuen.2018.03.007

[8] Lawlor DA, Harbord RM, Sterne JA, Timpson N, Davey SG. Mendelian randomization: Using genes as instruments for making causal inferences in epidemiology. Stat. Med. 2008;27(8):1133–1163. 17886233 10.1002/sim.3034

[9] Wu S, An S, Li W, Lichtenstein AH, Gao J, Kris-Etherton PM, Wu Y, Jin C, Huang S, Hu FB, et al. Association of trajectory of cardiovascular health score and incident cardiovascular disease. JAMA Netw. Open. 2019;2(5): Article e194758. 31150075 10.1001/jamanetworkopen.2019.4758 PMC6547110

[10] Yu Y, Dong Z, Li Y, Zhang J, Yin S, Gao X, Wu S, KaiLuan Study Investigators. The cardiovascular and cerebrovascular health in North China from 2006 to 2011: Results from the KaiLuan study. Front Cardiovasc Med. 2021;8: Article 683416. 34322527 10.3389/fcvm.2021.683416 PMC8310945

[11] Sun J, Hui Y, Li J, Zhao X, Chen Q, Li X, Wu N, Xu M, Liu W, Li R, et al. Protocol for multi-modality MEdical imaging sTudy bAsed on KaiLuan study (META-KLS): Rationale, design and database building. BMJ Open. 2023;13(2): Article e67283. 10.1136/bmjopen-2022-067283 PMC9923283 36764715

[12] Zhang Y, Pletcher MJ, Vittinghoff E, Clemons AM, Jacobs DR Jr, Allen NB, Alonso A, Bellows BK, Oelsner EC, Zeki al Hazzouri A, et al. Association between cumulative low-density lipoprotein cholesterol exposure during young adulthood and middle age and risk of cardiovascular events. JAMA Cardiol. 2021;6(12):1406. 34550307 10.1001/jamacardio.2021.3508 PMC8459309

[13] Schmidt P. Bayesian inference for structured additive regression models for large-scale problems with applications to medical imaging [thesis]. [München, Germany]: Ludwig-Maximilians Universitat Munchen; 2017.

[14] Alexander AL, Hurley SA, Samsonov AA, Adluru N, Hosseinbor AP, Mossahebi P, Tromp DPM, Zakszewski E, Field AS. Characterization of cerebral white matter properties using quantitative magnetic resonance imaging stains. Brain Connect. 2011;1(6):423–446. 22432902 10.1089/brain.2011.0071 PMC3360545

[15] Rosas HD, Lee SY, Bender AC, Zaleta AK, Vangel M, Yu P, Fischl B, Pappu V, Onorato C, Cha JH, et al. Altered white matter microstructure in the corpus callosum in Huntington’s disease: Implications for cortical “disconnection”. Neuroimage. 2010;49(4):2995–3004. 19850138 10.1016/j.neuroimage.2009.10.015 PMC3725957

[16] Weinstein G, Zelber-Sagi S, Preis SR, Beiser AS, DeCarli C, Speliotes EK, Satizabal CL, Vasan RS, Seshadri S. Association of nonalcoholic fatty liver disease with lower brain volume in healthy middle-aged adults in the Framingham study. JAMA Neurol. 2018;75(1):97–104. 29159396 10.1001/jamaneurol.2017.3229 PMC5833484

[17] Greenland S. An introduction to instrumental variables for epidemiologists. Int. J. Epidemiol. 2018;47(1):358. 29294084 10.1093/ije/dyx275

[18] Didelez V, Sheehan N. Mendelian randomization as an instrumental variable approach to causal inference. Stat. Methods Med. Res. 2007;16(4):309–330. 17715159 10.1177/0962280206077743

[19] Yengo L, Sidorenko J, Kemper KE, Zheng Z, Wood AR, Weedon MN, Frayling TM, Hirschhorn J, Yang J, Visscher PM, et al. Meta-analysis of genome-wide association studies for height and body mass index in ∼700000 individuals of European ancestry. Hum. Mol. Genet. 2018;27(20):3641–3649. 30124842 10.1093/hmg/ddy271 PMC6488973

[20] Smith SM, Douaud G, Chen W, Hanayik T, Alfaro-Almagro F, Sharp K, Elliott LT. An expanded set of genome-wide association studies of brain imaging phenotypes in UK Biobank. Nat. Neurosci. 2021;24(5):737–745. 33875891 10.1038/s41593-021-00826-4 PMC7610742

[21] Burgess S, Butterworth A, Thompson SG. Mendelian randomization analysis with multiple genetic variants using summarized data. Genet. Epidemiol. 2013;37(7):658–665. 24114802 10.1002/gepi.21758 PMC4377079

[22] Burgess S, Thompson SG. Interpreting findings from Mendelian randomization using the MR-Egger method. Eur. J. Epidemiol. 2017;32(5):377–389. 28527048 10.1007/s10654-017-0255-x PMC5506233

[23] Bowden J, Hemani G, Davey SG. Invited commentary: Detecting individual and global horizontal pleiotropy in Mendelian randomization—A job for the humble heterogeneity statistic? Am. J. Epidemiol. 2018;187(12):2681–2685. 30188969 10.1093/aje/kwy185 PMC6269239

[24] Verbanck M, Chen CY, Neale B, Do R. Detection of widespread horizontal pleiotropy in causal relationships inferred from Mendelian randomization between complex traits and diseases. Nat. Genet. 2018;50(5):693–698. 29686387 10.1038/s41588-018-0099-7 PMC6083837

[25] von Elm E, Altman DG, Egger M, Pocock SJ, Gøtzsche PC, Vandenbroucke JP. The strengthening the reporting of observational studies in epidemiology (STROBE) statement: Guidelines for reporting observational studies. Int. J. Surg.. 2014;12(4):1495–1499. 25046131 10.1016/j.ijsu.2014.07.013

[26] Skrivankova VW, Richmond RC, Woolf B, Davies NM, Swanson SA, VanderWeele TJ, Timpson NJ, Higgins JPT, Dimou N, Langenberg C, et al. Strengthening the reporting of observational studies in epidemiology using mendelian randomisation (STROBE-MR): Explanation and elaboration. BMJ. 2021;375: Article n2233. 34702754 10.1136/bmj.n2233 PMC8546498

[27] Gorelick PB, Furie KL, Iadecola C, Smith EE, Waddy SP, Lloyd-Jones DM, Bae HJ, Bauman MA, Dichgans M, Duncan PW, et al. Defining optimal brain health in adults: A presidential advisory from the American Heart Association/American Stroke Association. Stroke. 2017;48(10):e284–e303. 28883125 10.1161/STR.0000000000000148 PMC5654545

[28] Dekkers IA, Jansen PR, Lamb HJ. Obesity, brain volume, and white matter microstructure at MRI: A cross-sectional UK Biobank study. Radiology. 2019;291(3):763–771. 31012815 10.1148/radiol.2019181012

[29] García-García I, Michaud A, Jurado MÁ, Dagher A, Morys F. Mechanisms linking obesity and its metabolic comorbidities with cerebral grey and white matter changes. Rev. Endocr. Metab. Disord. 2022;23(4):833–843. 35059979 10.1007/s11154-021-09706-5

[30] Mole JP, Subramanian L, Bracht T, Morris H, Metzler-Baddeley C, Linden DE. Increased fractional anisotropy in the motor tracts of Parkinson’s disease suggests compensatory neuroplasticity or selective neurodegeneration. Eur. Radiol. 2016;26(10):3327–3335. 26780637 10.1007/s00330-015-4178-1 PMC5021738

[31] Franz CE, Xian H, Lew D, Hatton SN, Puckett O, Whitsel N, Beck A, Dale AM, Fang B, Fennema-Notestine C, et al. Body mass trajectories and cortical thickness in middle-aged men: A 42-year longitudinal study starting in young adulthood. Neurobiol. Aging. 2019;79:11–21. 31026618 10.1016/j.neurobiolaging.2019.03.003 PMC6591047

[32] Kim KW, Seo H, Kwak MS, Kim D. Visceral obesity is associated with white matter hyperintensity and lacunar infarct. Int. J. Obes. (Lond). 2017;41(5):683–688. 28104915 10.1038/ijo.2017.13

[33] Han YP, Tang X, Han M, Yang J, Cardoso MA, Zhou J, Simó R. Relationship between obesity and structural brain abnormality: Accumulated evidence from observational studies. Ageing Res. Rev. 2021;71: Article 101445. 34391946 10.1016/j.arr.2021.101445

[34] Cole JH, Ritchie SJ, Bastin ME, Valdés Hernández MC, Muñoz Maniega S, Royle N, Corley J, Pattie A, Harris SE, Zhang Q, et al. Brain age predicts mortality. Mol. Psychiatry. 2018;23(5):1385–1392. 28439103 10.1038/mp.2017.62 PMC5984097

[35] Cole JH, Poudel R, Tsagkrasoulis D, Caan MWA, Steves C, Spector TD, Montana G. Predicting brain age with deep learning from raw imaging data results in a reliable and heritable biomarker. Neuroimage. 2017;163:115–124. 28765056 10.1016/j.neuroimage.2017.07.059

[36] Cai H, Gao Y, Liu M. Graph transformer geometric learning of brain networks using multimodal MR images for brain age estimation. IEEE Trans. Med. Imaging. 2023;42(2):456–466. 36374874 10.1109/TMI.2022.3222093

[37] Bashyam VM, Erus G, Doshi J, Habes M, Nasrallah IM, Truelove-Hill M, Srinivasan D, Mamourian L, Pomponio R, Fan Y, et al. MRI signatures of brain age and disease over the lifespan based on a deep brain network and 14 468 individuals worldwide. Brain. 2020;143(7):2312–2324. 32591831 10.1093/brain/awaa160 PMC7364766

[38] Pan Y, Shen J, Cai X, Chen H, Zong G, Zhu W, Jing J, Liu T, Jin A, Wang Y, et al. Adherence to a healthy lifestyle and brain structural imaging markers. Eur. J. Epidemiol. 2023;38(6):657–668. 37060500 10.1007/s10654-023-00992-8

[39] Chen W, Feng J, Guo J, Dong S, Li R, NGO JCK, Wang C, Ma Y, Dong Z. Obesity causally influencing brain cortical structure: A Mendelian randomization study. Cereb. Cortex. 2023;33(15):9409–9416. 37328935 10.1093/cercor/bhad214

[40] Debette S, Wolf C, Lambert J, Crivello F, Soumaré A, Zhu YC, Schilling S, Dufouil C, Mazoyer B, Amouyel P, et al. Abdominal obesity and lower gray matter volume: A Mendelian randomization study. Neurobiol. Aging. 2014;35(2):378–386. 23998998 10.1016/j.neurobiolaging.2013.07.022

