# Supplementary material for: Association between Body Mass Index and Brain Health in Adults: A 16-Year Population-Based Cohort and Mendelian Randomization Study
Source: Health Data Sci. 2024 Mar 15;4:0087. doi: 10.34133/hds.0087 (PMC10944701; doi:10.34133/hds.0087)
Supplement: Supplementary 1 — Tables S1 to S7 [file hds.0087.f1.docx]

## Supplementary Materials

**Measurements of clinical features**

Demographic characteristics and results of clinical and laboratory examinations were collected during each visit based on the KaiLuan study. Clinical history was evaluated by trained doctors during their visits to the hospital. A history of hypertension was defined as a self-reported history of clinically diagnosed hypertension, use of antihypertensive medications, and/or systolic blood pressure ≥140 mmHg or diastolic blood pressure ≥90 mmHg during any visit. Since systolic and diastolic blood pressure were highly correlated at each visit (r=0.64 to 0.83, *p*<0.001), we only used systolic blood pressure as a covariate in this study. Any history of diabetes was defined as a self-reported history of clinical diagnosis of diabetes, the use of antidiabetic medications, or a fasting glucose level of ≥7.0 mmol/L during any visit. “Usually performing physical activity” was defined as participants who performed physical activity more than three times a week for 30 min per session. Participants were otherwise defined as “sometimes or seldom performing physical activity.”

Sex, smoking, alcohol consumption, physical activity habits, and diabetes were categorical variables. Continuous variables, including systolic blood pressure, triglycerides, high-density lipoprotein, and low-density lipoprotein, were calculated as cumulative values to reduce random errors.

**Supplementary Table 1.** Neuroimaging acquisition parameters

| **Sequence** | **Mode** | **Readout module** | **Time (min:s)** | **TR/TE (ms)** | **Bandwith (KHz)** | **Flip angle (degrees)** | **Number of slices** | **Slice thickness (mm)** | **Spacing between slices (mm)** | **FOV (cm^2^)** | **Acquisition matrix** | **Recon matrix** | **ETL** | **NEX** |
| --- | --- | --- | --- | --- | --- | --- | --- | --- | --- | --- | --- | --- | --- | --- |
| DTI | 2D | EPI | 4:24 | 8000/97.9 | 250 | 90 | 29 | 5 | 5 | 24×24 | 128×130 | 256×256 |  | 2 |
| 3D-BRAVO T1WI | 3D | GRE | 2:34 | 6.7/2.6 | 41.67 | 15 | 170 | 1 | 1 | 25.6×25.6 | 256×256 | 512×512 |  | 1 |
| FLAIR | 3D | FSE | 6:23 | 5000/1147 | 62.5 | 90 | 170 | 1 | 1 | 25.6×25.6 | 256×256 | 512×512 | 160 | 1 |
| T2WI | 2D | FSE | 1:27 | 5842/103 | 62.5 | 142 | 25 | 5 | 6 | 24×24 | 416×416 | 512×512 | 32 | 1.5 |
| DWI | 2D | EPI | 0:38 | 5110/77.2 | 250 | 90 | 25 | 5 | 6 | 24×24 | 130×160 | 256×256 |  | 2 |

DTI, diffusion tensor imaging; 3D-BRAVO T1WI, three-dimensional brain volume T1-weighted image; FLAIR, fluid-attenuated inversion recovery; SWAN, susceptibility-weighted angiography; T2WI, T2-weighted image; DWI, diffusion-weighted imaging; FSE, fast spin echo; GRE, gradient-recalled echo; EPI, echo-planar imaging; TR, repetition time; TE, echo time; FOV, field of view; ETL, echo train length; NEX, number of excitations.

DTI was set to acquire 15 directions, with b=1,000 mm^2^/s.

**Supplementary Table 2.** Pleiotropy and heterogeneity tests using Cochran’s Q tests

| **Outcome** | **Pleiotropy test** | | | **Cochran’s Q test** | |
| --- | --- | --- | --- | --- | --- |
|  | **Egger-intercept** | **SE** | **p-value** | **Cochran’s Q** | **p-value** |
| Brain volume | 8.35E-04 | 1.29E-03 | 5.16E-01 | 715.4577282 | 3.42E-11 |
| Gray matter volume | 5.12E-04 | 1.25E-03 | 6.82E-01 | 684.7056084 | 6.44E-09 |
| White matter volume | -5.48E-04 | 1.26E-03 | 6.65E-01 | 697.5257409 | 5.16E-10 |
| Cerebrospinal fluid volume | 8.73E-04 | 1.24E-03 | 4.82E-01 | 654.7170025 | 2.08E-07 |
| White matter hyperintensity volume | 3.82E-04 | 1.19E-03 | 7.48E-01 | 604.7606344 | 2.65E-04 |
| Left hippocampus volume | 4.50E-04 | 1.15E-03 | 6.96E-01 | 585.8331309 | 1.51E-03 |
| Right hippocampus volume | -6.36E-04 | 1.12E-03 | 5.71E-01 | 549.3621626 | 2.26E-02 |
| FA in pontine crossing tract | 3.23E-04 | 1.18E-03 | 7.84E-01 | 582.520855 | 2.26E-03 |
| FA in middle cerebellar peduncle | 9.04E-04 | 1.23E-03 | 4.64E-01 | 630.3053688 | 9.01E-06 |
| FA in left corticospinal tract | -9.77E-04 | 1.19E-03 | 4.14E-01 | 601.3422631 | 4.20E-04 |
| FA in right corticospinal tract | -8.90E-04 | 1.22E-03 | 4.65E-01 | 623.0440809 | 4.12E-05 |
| MD in forceps minor tract | -8.24E-04 | 1.14E-03 | 4.72E-01 | 540.7670223 | 3.76E-02 |
| MD in tract left inferior fronto-occipital fasciculus | -2.72E-04 | 1.12E-03 | 8.08E-01 | 522.4156527 | 1.29E-01 |
| MD in right inferior fronto-occipital fasciculus tract | -4.00E-04 | 1.16E-03 | 7.29E-01 | 556.8225313 | 1.54E-02 |
| MD in left anterior thalamic radiation tract | -3.49E-04 | 1.17E-03 | 7.66E-01 | 567.0098922 | 5.38E-03 |
| MD in right uncinate fasciculus | 4.50E-04 | 1.21E-03 | 7.10E-01 | 608.0213963 | 1.68E-04 |

SE, standard error; CSF, cerebrospinal fluid; FA, fractional anisotropy; MD, mean diffusivity. Cochran’s Q statistics were calculated using the inverse-variance weighted (IVW) method.

**Supplementary Table 3.** Sensitivity analysis of the causal association estimation of BMI on neuroimaging features

| **MR methods** | **Brain volume** | | | | | | **Gray matter volume** | | | | **White matter volume** | | | |
| --- | --- | --- | --- | --- | --- | --- | --- | --- | --- | --- | --- | --- | --- | --- |
|  | **SNP(N)** | **β** | | **SE** | | **p-value** | **SNP(N)** | **β** | **SE** | **p-value** | **SNP(N)** | **β** | **SE** | **p-value** |
| Inverse-variance weighted | 485 | -7.46E-02 | | 2.98E-02 | | **1.23E-02** | 487 | -8.65E-02 | 2.91E-02 | **2.94E-03** | 484 | -1.28E-02 | 2.95E-02 | 6.64E-01 |
| MR Egger | 485 | -1.23E-01 | | 7.95E-02 | | 1.24E-01 | 487 | -1.16E-01 | 7.73E-02 | 1.35E-01 | 484 | 1.85E-02 | 7.79E-02 | 8.13E-01 |
| Weighted median | 485 | -4.80E-02 | | 4.25E-02 | | 2.59E-01 | 487 | -9.66E-02 | 4.78E-02 | **4.33E-02** | 484 | 1.11E-02 | 4.27E-02 | 7.94E-01 |
| Simple mode | 485 | -8.23E-03 | | 1.41E-01 | | 9.54E-01 | 487 | -1.11E-01 | 1.37E-01 | 4.19E-01 | 484 | -2.26E-02 | 1.43E-01 | 8.75E-01 |
| Weighted mode | 485 | -4.17E-02 | | 7.84E-02 | | 5.95E-01 | 487 | -1.65E-01 | 7.41E-02 | **2.65E-02** | 484 | -9.97E-03 | 8.10E-02 | 9.02E-01 |
|  |  |  | |  | |  |  |  |  |  |  |  |  |  |
| (continued table) | **Cerebrospinal fluid volume** | | | | | | **Left hippocampus volume** | | | | **Right hippocampus volume** | | | |
|  | **SNP(N)** | **β** | | **SE** | | **p-value** | **SNP(N)** | **β** | **SE** | **p-value** | **SNP(N)** | **β** | **SE** | **p-value** |
|  | 482 | -2.96E-02 | | 2.87E-02 | | 3.03E-01 | 489 | -1.35E-02 | 2.68E-02 | 6.14E-01 | 486 | -3.21E-03 | 2.62E-02 | 9.02E-01 |
|  | 482 | -7.97E-02 | | 7.67E-02 | | 2.99E-01 | 489 | -3.92E-02 | 7.11E-02 | 5.81E-01 | 486 | 3.33E-02 | 6.94E-02 | 6.32E-01 |
|  | 482 | -6.33E-03 | | 4.49E-02 | | 8.88E-01 | 489 | -6.54E-02 | 4.15E-02 | 1.15E-01 | 486 | -1.13E-02 | 4.14E-02 | 7.85E-01 |
|  | 482 | 1.12E-01 | | 1.41E-01 | | 4.28E-01 | 489 | -9.29E-02 | 1.29E-01 | 4.72E-01 | 486 | 1.32E-01 | 1.27E-01 | 2.99E-01 |
|  | 482 | 4.18E-02 | | 8.22E-02 | | 6.12E-01 | 489 | -1.27E-01 | 8.27E-02 | 1.24E-01 | 486 | -2.24E-02 | 7.72E-02 | 7.72E-01 |
|  |  |  | |  | |  |  |  |  |  |  |  |  |  |
| (continued table) | **White matter hyperintensity volume** | | | | | | **FA in pontine crossing tract** | | | | **FA in middle cerebellar peduncle** | | | |
|  | **SNP(N)** | **β** | | **SE** | | **p-value** | **SNP(N)** | **β** | **SE** | **p-value** | **SNP(N)** | **β** | **SE** | **p-value** |
|  | 490 | 1.62E-02 | | 2.76E-02 | | 5.57E-01 | 490 | 4.45E-02 | 2.74E-02 | 1.04E-01 | 486 | 7.34E-02 | 2.87E-02 | **1.06E-02** |
|  | 490 | -5.66E-03 | | 7.34E-02 | | 9.39E-01 | 490 | 2.61E-02 | 7.28E-02 | 7.20E-01 | 486 | 2.17E-02 | 7.62E-02 | 7.76E-01 |
|  | 490 | 3.13E-02 | | 4.43E-02 | | 4.80E-01 | 490 | -7.02E-03 | 4.62E-02 | 8.79E-01 | 486 | 8.04E-02 | 4.88E-02 | 9.94E-02 |
|  | 490 | 6.29E-03 | | 1.28E-01 | | 9.61E-01 | 490 | -3.96E-02 | 1.37E-01 | 7.73E-01 | 486 | 8.85E-02 | 1.55E-01 | 5.69E-01 |
|  | 490 | 3.84E-02 | | 6.77E-02 | | 5.70E-01 | 490 | -6.00E-02 | 7.49E-02 | 4.23E-01 | 486 | 2.23E-02 | 9.82E-02 | 8.21E-01 |
|  |  |  |  | |  | |  |  |  |  |  |  |  |  |
| (continued table) | **FA in left corticospinal tract** | | | | | | **FA in right corticospinal tract** | | | | **MD in forceps minor tract** | | | |
|  | **SNP(N)** | **β** | | **SE** | | **p-value** | **SNP(N)** | **β** | **SE** | **p-value** | **SNP(N)** | **β** | **SE** | **p-value** |
|  | 491 | 6.84E-02 | | 2.78E-02 | | **1.39E-02** | 491 | 4.75E-02 | 2.83E-02 | 9.31E-02 | 485 | -4.17E-03 | 2.67E-02 | 8.76E-01 |
|  | 491 | 1.24E-01 | | 7.38E-02 | | 9.28E-02 | 491 | 9.85E-02 | 7.51E-02 | 1.91E-01 | 485 | 4.30E-02 | 7.07E-02 | 5.44E-01 |
|  | 491 | 1.05E-01 | | 4.45E-02 | | **1.87E-02** | 491 | 5.15E-02 | 4.44E-02 | 2.46E-01 | 485 | 3.84E-02 | 4.22E-02 | 3.63E-01 |
|  | 491 | -1.31E-01 | | 1.38E-01 | | 3.45E-01 | 491 | -4.22E-02 | 1.53E-01 | 7.84E-01 | 485 | -2.48E-01 | 1.32E-01 | 6.06E-02 |
|  | 491 | 1.57E-01 | | 8.46E-02 | | 6.46E-02 | 491 | 1.13E-01 | 9.79E-02 | 2.47E-01 | 485 | 1.59E-01 | 8.15E-02 | 5.24E-02 |

| (continued table) | **MD in left inferior fronto-occipital fasciculus tract** | | | | | **MD in right inferior fronto-occipital fasciculus tract** | | | | **MD in left anterior thalamic radiation tract** | | | |
| --- | --- | --- | --- | --- | --- | --- | --- | --- | --- | --- | --- | --- | --- |
|  | **SNP(N)** | **β** | **SE** | | **p-value** | **SNP(N)** | **β** | **SE** | **p-value** | **SNP(N)** | **β** | **SE** | **p-value** |
|  | 488 | -3.02E-02 | 2.61E-02 | | 2.46E-01 | 488 | -3.42E-02 | 2.69E-02 | 2.03E-01 | 485 | -4.72E-02 | 2.73E-02 | 8.38E-02 |
|  | 488 | -1.46E-02 | 6.92E-02 | | 8.33E-01 | 488 | -1.13E-02 | 7.14E-02 | 8.74E-01 | 485 | -2.73E-02 | 7.23E-02 | 7.06E-01 |
|  | 488 | 1.37E-02 | 4.41E-02 | | 7.56E-01 | 488 | 2.81E-02 | 4.50E-02 | 5.32E-01 | 485 | -3.42E-02 | 4.28E-02 | 4.24E-01 |
|  | 488 | 9.33E-03 | 1.42E-01 | | 9.47E-01 | 488 | -2.64E-02 | 1.35E-01 | 8.45E-01 | 485 | -2.19E-01 | 1.29E-01 | 9.02E-02 |
|  | 488 | 7.16E-02 | 7.62E-02 | | 3.48E-01 | 488 | 5.80E-02 | 7.87E-02 | 4.62E-01 | 485 | 2.58E-02 | 7.52E-02 | 7.32E-01 |
|  |  |  | |  |  |  |  |  |  |  |  |  |  |
| (continued table) | **MD in right uncinate fasciculus** | | | | |  |  |  |  |  |  |  |  |
|  | **SNP(N)** | **β** | **SE** | | **p-value** |  |  |  |  |  |  |  |  |
|  | 489 | -3.89E-02 | 2.81E-02 | | 1.66E-01 |  |  |  |  |  |  |  |  |
|  | 489 | -6.47E-02 | 7.46E-02 | | 3.87E-01 |  |  |  |  |  |  |  |  |
|  | 489 | -3.12E-03 | 4.29E-02 | | 9.42E-01 |  |  |  |  |  |  |  |  |
|  | 489 | 1.61E-02 | 1.31E-01 | | 9.02E-01 |  |  |  |  |  |  |  |  |
|  | 489 | 5.77E-02 | 8.31E-02 | | 4.88E-01 |  |  |  |  |  |  |  |  |

MR, Mendelian randomisation; SNP, single-nucleotide polymorphism; N, number; SE, standard error; CSF, cerebrospinal fluid; FA, fractional anisotropy; MD, mean diffusivity.

**Supplementary Table 4. Association of cumulative BMI with brain macrostructural volume, brain microstructural integrity, and white matter hyperintensity, further adjusted for cognitive evaluation**

| **Neuroimaging Features** | **Cumulative BMI, kg/m^2^** | | | |
| --- | --- | --- | --- | --- |
|  | **Low** | **Medium** | **High** | ***p* Value** |
|  | **(<23.56)** | **(23.56-26.20)** | **(>26.20)** | **for Trend** |
| **Cumulative BMI, mean (SD), kg/m^2^** | 21.8(1.3)  N=357 | 24.9(0.8)  N=353 | 28.3(1.7)  N=364 |  |
| **Relative brain macrostructural volume, % of TIV** |  |  |  |  |
| Cerebral parenchyma | 0(ref) | -0.20(-0.60 to 0.20) | **-0.60(-1.0 to -0.10)** | **0.038** |
| Gray matter | 0(ref) | -0.30(-0.60 to 0.00) | **-0.50(-0.80 to -0.20)** | **0.006** |
| White matter | 0(ref) | 0.10(-0.20 to 0.40) | -0.10(-0.40 to 0.20) | 0.322 |
| Cerebrospinal fluid | 0(ref) | 0.20(-0.20 to 0.60) | **0.60(0.10 to 1.00)** | **0.036** |
| Hippocampus | 0(ref) | -1.0E-4(-3.0E-3 to 3.2E-3) | -1.6E-3(-5.1E-3 to 1.8E-3) | 0.505 |
| **Brain microstructural integrity** |  |  |  |  |
| Fractional anisotropy | 0(ref) | 0.003(-0.001 to 0.006) | 0.002(-0.002 to 0.006) | 0.348 |
| Mean diffusivity, 10^-3^ mm^2^/s | 0(ref) | -0.003(-0.008 to 0.002) | -0.003(-0.008 to 0.002) | 0.389 |
| **Relative white matter hyperintensity volume, % of TIV** |  |  |  |  |
| White matter hyperintensity | 0(ref) | 0(-0.10 to 0.10) | **0.20(0.00 to 0.30)** | **0.011** |
| Periventricular white matter hyperintensity | 0(ref) | 0.01(0.00 to 0.10) | **0.10(0.00 to 0.10)** | **0.017** |
| Deep white matter hyperintensity | 0(ref) | 0(-0.10 to 0.10) | **0.10(0.01 to 0.20)** | **0.017** |

Abbreviations: BMI, body mass index; TIV, total intracranial volume; IQR, interquartile range; CI, confidence interval

Results were further adjusted for cognitive evaluation, in addition to Model 2 in Table 2. Results are presented as β (95% CI). Values are presented as mean (SD), median (IQR), or no. (%).

**Supplementary Table 5. Association of cumulative BMI with brain macrostructural volume, brain microstructural integrity, and white matter hyperintensity stratified by age, further adjusted for cognitive evaluation**

| **Neuroimaging features** | **Age<45 yrs** | | | | **45 yrs≤Age<60 yrs** | | | | **Age≥60 yrs** | | | |
| --- | --- | --- | --- | --- | --- | --- | --- | --- | --- | --- | --- | --- |
|  | **Low (<23.56)** | **Medium (23.56-26.20)** | **High (>26.20)** | ***p* Value for Trend** | **Low (<23.56)** | **Medium (23.56-26.20)** | **High (>26.20)** | ***p* Value for Trend** | **Low (<23.56)** | **Medium (23.56-26.20)** | **High (>26.20)** | ***p* Value for Trend** |
| **Cumulative BMI, mean (SD), kg/m^2^** | 21.5(1.3) | 24.8(0.8) | 28.8(1.9) |  | 21.8(1.2) | 24.9(0.8) | 28.1(1.7) |  | 22.1(1.3) | 24.8(0.8) | 28.2(1.6) |  |
| **Relative brain macrostructural volume, % of TIV** |  |  |  |  |  |  |  |  |  |  |  |  |
| Cerebral parenchyma | 0(ref) | 0.50(-0.30 to 1.30) | **-1.10(-2.00 to -0.30)** | **0.001** | 0(ref) | -0.50(-1.20 to 0.10) | -0.40(-1.10 to 0.30) | 0.223 | 0(ref) | -0.50(-1.20 to 0.20) | -0.60(-1.40 to 0.20) | 0.292 |
| Gray matter | 0(ref) | 0.40(-0.20 to 1.00) | **-0.80(-1.50 to -0.20)** | **0.001** | 0(ref) | -0.50(-0.90 to -0.10) | -0.50(-0.90 to 0.00) | 0.070 | 0(ref) | -0.50(-1.00 to -0.10) | -0.30(-0.80 to 0.20) | 0.091 |
| White matter | 0(ref) | 0.10(-0.40 to 0.70) | -0.30(-0.80 to 0.30) | 0.450 | 0(ref) | -0.10(-0.50 to 0.40) | 0(-0.40 to 0.50) | 0.870 | 0(ref) | 0(-0.40 to 0.50) | -0.30(-0.80 to 0.20) | 0.326 |
| Cerebrospinal fluid | 0(ref) | -0.50(-1.30 to 0.30) | **1.10(0.20 to 1.90)** | **0.001** | 0(ref) | 0.60(-0.10 to 1.20) | 0.40(-0.30 to 1.10) | 0.198 | 0(ref) | 0.50(-0.20 to 1.20) | 0.60(-0.20 to 1.40) | 0.262 |
| Hippocampus | 0(ref) | 2.6E-3(-3.3E-3 to 8.6E-3) | -4.0E-3(0.00 to 2.1E-3) | 0.117 | 0(ref) | -1.6E-3(-6.1E-3 to 2.9E-3) | -6.7E-4(-5.2E-3 to 5.0E-3) | 0.679 | 0(ref) | -1.9E-3(-7.2E-3 to 3.4E-3) | -2.2E-3(-8.1E-3 to 3.8E-3) | 0.727 |
| **Brain microstructural integrity** |  |  |  |  |  |  |  |  |  |  |  |  |
| Fractional anisotropy | 0(ref) | 0.003(-0.002 to 0.008) | -0.001(-0.006 to 0.004) | 0.360 | 0(ref) | -0.001(-0.004 to 0.003) | 0.003(-0.001 to 0.008) | 0.077 | 0(ref) | 0.003(-0.005 to 0.010) | 0(-0.009 to 0.009) | 0.701 |
| Mean diffusivity, 10^-3^ mm^2^/s | 0(ref) | -0.007(-0.014 to 0) | 0(-0.007 to 0.007) | 0.119 | 0(ref) | 0.003(-0.003 to 0.009) | -0.003(-0.009 to 0.004) | 0.133 | 0(ref) | -0.001(-0.011 to 0.008) | 0(-0.009 to 0.011) | 0.899 |
| **Relative white matter hyperintensity volume, % of TIV** |  |  |  |  |  |  |  |  |  |  |  |  |
| White matter hyperintensity | 0(ref) | 0(-0.10 to 0.10) | 0.10(0.01 to 0.20) | 0.086 | 0(ref) | -0.10(-0.20 to 0.00) | 0.10(-0.10 to 0.20) | 0.035 | 0(ref) | 0.20(-0.10 to 0.50) | 0.40(0.10 to 0.70) | 0.054 |
| Periventricular white matter hyperintensity | 0(ref) | 0(0.00 to 0.00) | **0.10(0.00 to 0.10)** | **0.003** | 0(ref) | 0(-0.10 to 0.00) | 0(0.00 to 0.10) | 0.232 | 0(ref) | **0.10(0.00 to 0.20)** | **0.20(0.00 to 0.30)** | **0.043** |
| Deep white matter hyperintensity | 0(ref) | 0(-0.10 to 0.10) | 0.10(0.00 to 0.10) | 0.337 | 0(ref) | -0.10(-0.10 to 0.00) | 0(-0.10 to 0.10) | 0.022 | 0(ref) | 0.10(-0.10 to 0.30) | 0.20(0.00 to 0.40) | 0.097 |

Abbreviations: BMI, body mass index; TIV, total intracranial volume; CI, confidence interval

Results were further adjusted for cognitive evaluation, in addition to Model 2 in Table 2. Results are presented as β (95% CI).

**Supplementary Table 6. Association of cumulative BMI with brain macrostructural volume, brain microstructural integrity, and white matter hyperintensity, excluded subjects with BMI lower than 18.5 kg/m^2^**

| **Neuroimaging Features** | **Cumulative BMI, kg/m^2^** | | | |
| --- | --- | --- | --- | --- |
|  | **Low** | **Medium** | **High** | ***p* Value** |
|  | **(18.5-23.56)** | **(23.56-26.20)** | **(>26.20)** | **for Trend** |
| **Cumulative BMI, mean (SD), kg/m^2^** | 21.8(1.2)  N=352 | 24.9(0.8)  N=353 | 28.3(1.7)  N=364 |  |
| **Relative brain macrostructural volume, % of TIV** |  |  |  |  |
| Cerebral parenchyma | 0(ref) | -0.20(-0.60 to 0.20) | **-0.60(-1.11 to -0.20)** | **0.024** |
| Gray matter | 0(ref) | **-0.30(-0.60 to 0.10)** | **-0.50(-0.80 to -0.20)** | **0.002** |
| White matter | 0(ref) | 0.10(-0.20 to 0.40) | -0.10(-0.40 to 0.20) | 0.428 |
| Cerebrospinal fluid | 0(ref) | 0.20(-0.20 to 0.70) | **0.60(0.20 to 1.10)** | **0.023** |
| Hippocampus | 0(ref) | -2.7E-4(-3.4E-3 to 2.8E-3) | -1.8E-3(-5.2E-3 to 1.6E-3) | 0.505 |
| **Brain microstructural integrity** |  |  |  |  |
| Fractional anisotropy | 0(ref) | 0.003(-0.001 to 0.006) | 0.002(-0.002 to 0.006) | 0.285 |
| Mean diffusivity, 10^-3^ mm^2^/s | 0(ref) | -0.004(-0.009 to 0.001) | -0.003(-0.009 to 0.002) | 0.255 |
| **Relative white matter hyperintensity volume, % of TIV** |  |  |  |  |
| White matter hyperintensity | 0(ref) | 0(-0.10 to 0.10) | **0.20(0.00 to 0.30)** | **0.007** |
| Periventricular white matter hyperintensity | 0(ref) | 0.01(0.00 to 0.10) | **0.10(0.00 to 0.10)** | **0.009** |
| Deep white matter hyperintensity | 0(ref) | 0(-0.10 to 0.10) | **0.10(0.01 to 0.20)** | **0.013** |

Abbreviations: BMI, body mass index; TIV, total intracranial volume; IQR, interquartile range; CI, confidence interval

Results were fully adjusted, same as Model 2 in Table 2. Results are presented as β (95% CI). Values are presented as mean (SD), median (IQR), or no. (%).

**Supplementary Table 7. Association of cumulative BMI with brain macrostructural volume, brain microstructural integrity, and white matter hyperintensity, with cut-off values as 24.0 and 28.0 kg/m^2^ for three groups**

| **Neuroimaging Features** | **Cumulative BMI, kg/m^2^** | | | |
| --- | --- | --- | --- | --- |
|  | **Low** | **Medium** | **High** | ***p* Value** |
|  | **(<24.0)** | **(24.0-28.0)** | **(>28.0)** | **for Trend** |
| **Cumulative BMI, mean (SD), kg/m^2^** | 22.1(1.4)  N=428 | 25.9(1.1)  N=484 | 29.8(1.4)  N=162 |  |
| **Relative brain macrostructural volume, % of TIV** |  |  |  |  |
| Cerebral parenchyma | 0(ref) | -0.10(-0.50 to 0.20) | **-0.70(-1.20 to -0.10)** | **0.042** |
| Gray matter | 0(ref) | -0.30(-0.60 to -0.10) | **-0.60(-0.90 to -0.20)** | **0.003** |
| White matter | 0(ref) | 0.20(0.00 to 0.50) | -0.10(-0.50 to 0.30) | 0.092 |
| Cerebrospinal fluid | 0(ref) | 0.10(-0.20 to 0.50) | **0.70(0.10 to 1.20)** | **0.040** |
| Hippocampus | 0(ref) | -7.5E-4(-3.6E-3 to 2.1E-3) | -2.0E-3(-6.1E-3 to 2.0E-3) | 0.606 |
| **Brain microstructural integrity** |  |  |  |  |
| Fractional anisotropy | 0(ref) | 0.004(0 to 0.007) | 0.005(0 to 0.009) | 0.053 |
| Mean diffusivity, 10^-3^ mm^2^/s | 0(ref) | **-0.006(-0.010 to -0.001)** | -0.006(-0.012 to 0.001) | **0.031** |
| **Relative white matter hyperintensity volume, % of TIV** |  |  |  |  |
| White matter hyperintensity | 0(ref) | 0(-0.10 to 0.10) | **0.20(0.01 to 0.40)** | **0.007** |
| Periventricular white matter hyperintensity | 0(ref) | 0.01(0.00 to 0.10) | **0.10(0.00 to 0.10)** | **0.031** |
| Deep white matter hyperintensity | 0(ref) | 0.01(-0.10 to 0.10) | **0.10(0.00 to 0.20)** | **0.007** |

Abbreviations: BMI, body mass index; TIV, total intracranial volume; IQR, interquartile range; CI, confidence interval

Results were fully adjusted, same as Model 2 in Table 2. Results are presented as β (95% CI). Values are presented as mean (SD), median (IQR), or no. (%).
